# The Registry of Accredited Companies in the Construction Sector in Spain: An Administrative Instrument for Risk-Prevention Control

**DOI:** 10.3390/ijerph16173133

**Published:** 2019-08-28

**Authors:** Álvaro Romero-Barriuso, Blasa MaríaVillena-Escribano, María de las Nieves González-García, María Segarra-Cañamares, Ángel Rodríguez-Sáiz

**Affiliations:** 1 Departamento de Construcciones Arquitectónicas y su Control, Universidad Politécnica de Madrid, 28040 Madrid, Spain; 2 Department of Civil Engineering & Edification, University of Castile-La Mancha, 16071 Cuenca, Spain; 3 Department of Architectural Constructions & Construction Engineering and Land, University of Burgos, 09001 Burgos, Spain

**Keywords:** registry of accredited companies, prevention, law on subcontracting, public administration, construction, labor legislation

## Abstract

The degree of compliance with the *Registro* *de* *Empresas* *Acreditadas* (REA) (Registry of Accredited Companies) and its implementation by the Public Administrations in Spain is compared with its implementation among private construction sector firms. The Registry of Accredited Companies is a tool for risk-prevention control that is defined by Law 32/2006 in Regulation of Subcontracting in the Construction Sector in Spain. On the basis of a quantitative analysis of the data obtained from public bodies registered with the REA, the study is limited to *Ayuntamientos* *y* *Diputaciones* *Provinciales* (Municipal Town and City Councils and Provincial Councils of the Provincial Government). To do so, the registration records with the REA of both public administrations are analyzed within the 50 Provinces and the two Autonomous Cities that together constitute the 17 Autonomous Communities of the national territory of Spain. In parallel, a comparative study is performed of the registration records of private construction sector firms registered with the REA. Public digital data-management tools are used for the investigation, together with publicly available information known as the *Relación* *de* *Puestos* *de* *Trabajo* (RPT) (List of Employment Positions) of the corresponding public entities under analysis, with the objective of testing the information and validating its degree of reliability. Likewise, a survey is administered to gather data on the registration of private construction center firms, in addition to the use of the qualitative Focus Groups technique, so as to assure the reliability the survey data. The results revealed unequal treatment by the Labor Authority with regard to the imposition of similar administrative obligations. A clearly negative discrimination was noted with regard to private construction sector firms, in comparison with the permissive attitude and light administrative burden of the Public Administrations.

## 1. Introduction

Significant changes have affected the production systems of goods and services over time, at a technological, technical, and organizational level; all motivated by continuous innovation, the efficiency of manufacturing processes, and the search for better product quality at more competitive prices [1,2,3].

In the construction industry, production and organizational systems at work present unique aspects and sector-specific differences, in comparison with other industrial processes, such as the temporality of the contractual relation and the singularity of the constructed product [4]. Nevertheless, this Sector has also undergone significant strategic business changes at a global level, incorporating innovative productive models based on specialization, greater efficiency, and competitiveness, adding greater value to the final product [5].

Although there are various forms of collaboration between firms in construction activities, the subcontracting of part of the project to other firms continues to be standard practice [6,7]. Hence, participation in projects with various firms specialized in particular skills and with professional experts in different construction trades is habitual in the construction sector [8].

Moreover, changes to the productive systems of the construction sector have created schemes of organization in which most of the activity is outsourced to subcontracting firms, leaving the main firm relegated to the role of a mere administrator and manager of the construction process [9,10], prompting greater precariousness of employment [11].

As in other countries [10,11,12], most subcontracting firms from the construction sector in Spain are Small and Medium-Size Enterprises [13], or they are formed solely of self-employed workers (Table 1). Up until 2007, the uncontrolled development of construction in Spain, especially the construction of housing units [14], had been provoking significant imbalances in labor relations and in production systems, through the employment of unskilled mass labor and the use of traditional technologies that had yet to modernize [15], with neither investment in training and quality, nor in safety, innovation, and new technologies [16,17,18,19].

One of the main problems arising from this working practice, which has impacted more than any other on Spanish society, is the unprecedented increase of occupational accidents in the construction sector, where the statistical records throughout the years of economic development have been particularly worrying [21]. In fact, the production model, based on the subcontracting of additional activities that constitute the whole construction project, has produced a significant lack of control over processes and product quality, impacting on the business standing of subcontracting firms. The selection criteria in accordance with the lowest offer have left little room for investment both in the training of workers [22,23] and in safety and risk-prevention in the workplace.

### The Registry of Accredited Companies in the Construction Sector

In view of the worrying incidence rates in the construction sector in Spain, the Government and social agents, and both firms and workers, were in agreement with the enactment of Law 32/2006 [24] and its inclusion in the regulations on Occupational Risk-Prevention, to facilitate the management of subcontracting in construction works, through a series of objective limitations on subcontracting chains [25,26,27]. Subsequently, the Registry of Accredited Companies (REA) was designed and implemented through Royal Decree 1109/2007 [28], of August 24th, from which the regulations of Law 32/2006 were developed. In that way, a direct relation was established between accident rates and non-compliance with the REA. Among the first twenty causes of fatal accidents we can see “Non-compliance with work procedures and instructions”, according to data from the National Institute of Safety and Health at Work, which represented 1.30% of accidents that occur in the sector, according to data from 2015 [29].

Both registration with the REA and the Duty of Preventive Coordination of Business Activities are inherent obligations for firms that employ workers, as long as they are acting as either contractors or subcontractors of construction works, regardless of their public or private status.

The problem arises when the promoter of the works is a Public Administration and acts as a contractor, a very common circumstance in Spain, because the Autonomous Communities, the Provincial Councils, and the Municipal Councils have a staff of both white-collar and blue-collar public-sector employees. In addition, they plan and execute construction activities as contractors within the scope of their competences, whether regional, provincial, or local [30]. In this context, part of the construction project is subcontracted to other firms, so that those public authorities become *de facto* contractors, having the legal obligation, therefore, to register their details on the REA in the relevant Autonomous Region (Table 2).

The purpose of this study is to ascertain the degree of compliance of the Public Administrations in their roles as contractors in construction activities with regard to the legal requirement to register with the REA, in the same way as any other firm, having neither prerogatives nor exceptions, simply because they form part of a Public Administration. Levels of compliance with the obligation to register will likewise be compared between Public Administrations and private construction sector firms in Spain.

As stated above, the *Registro de Empresas Acreditadas* (REA) [Registry of Accredited Companies] is an administrative risk-prevention instrument of the Ministry of Employment and Social Security of the Government of Spain. It regulates levels of monitoring and administrative control of safety at work with the objective of guaranteeing that firms that operate in the construction sector in Spain comply with the requirements relating to capacity and quality in matters of Occupational Risk-Prevention (ORP), as contained in Royal Decree 1627/1997, of October 24th [31], establishing minimum requirements on safety and health in construction works [32].

This registry emerged as a consequence of the previously mentioned high incidence rate in the construction sector in Spain, especially during the so-called ‘real-estate bubble’ (1997–2006) and the subsequent economic and financial crisis (2007–2016), also known as the ‘economic slowdown’, establishing controls on risk prevention and employment in construction firms.

Weak controls over subcontracting represented a constraint on construction works and their productivity in Spain. The principal problem was the inexistence of limits in the subcontracting chain, which therefore exacerbated the precariousness of working conditions, especially with regard to training resources in risk-prevention and safety. This absence of control over the organizational system of the contracting chain accelerated the imperative need to demand compliance with acceptable quality and solvency requirements from subcontracting firms with the requirements on capacity and quality in matters concerning ORP.

Although the requirements demanded by the REA were already set down in the Spanish regulations [31,33], the novelty arose when establishing the obligation to “accredit them through documentation with the contracting firm”, as well as with the Labor Authority. The Labor Inspectorate of the Ministry of Employment and Social Security may likewise request the documentation and the Certificate of Registration with the REA at any time.

As a guarantee, temporary updating of the register was introduced, because the entry on the REA is only valid over a period of three calendar years. Once that time has elapsed, the firms should renew their registration, providing the required documentation updated on the date of renewal.

Moreover, a series of requirements were established, as a *sine qua non* condition, so that firms could register and operate as contractors or subcontractors in construction activities in Spain.

**Figure 1:**
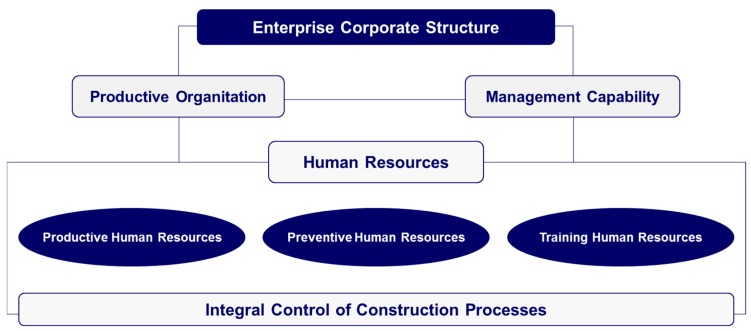
Requirements established in the REA for the accreditation of both the human and the material resources of subcontracting firms.

Contracting and subcontracting firms must provide documentary evidence that they have an organizational structure, with the necessary human and material resources, and the required solvency, to be able to manage the contracted works and to complete them (Figure 1). Likewise, they must assume the risk-prevention management of construction-related activities, in accordance with the obligations and responsibilities of entrepreneurial activity.

In addition to production-related, material, and human factors necessary for the development of the works, a training structure is required in matters concerning ORP (Figure 1), providing evidence of the presence of professionals for training construction workers in the management of risks associated with their activities. This risk-prevention training should be accredited by an authorized body, such as an External Risk-Prevention Service or by the Labor Construction Foundation (a private sector organization launched in 1992 by entrepreneurs and construction unions) for the validation of safety and training courses in the construction sector.

**Figure 2:**
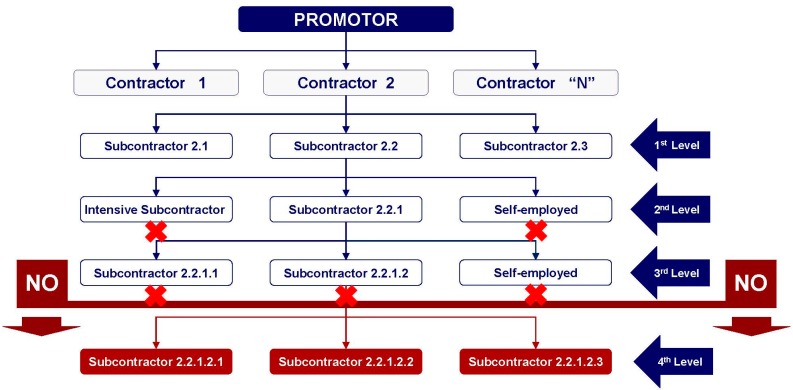
Restrictions on the maximum permitted levels in the subcontracting chain within construction works laid down by the Law on Subcontracting. *Source:* Ley 32/2006, reguladora de la subcontratación en el Sector de la Construcción [24].

It was moreover decided to limit the possibility of subcontracting from the Third Level (Figure 2), so as to avoid excessive numbers of subcontracted firms, except under justified circumstances or in unforeseen situations, which would require the presence of a specialized firm for technical reasons.

Registration on the REA is not mandatory for self-employed workers with no salaried workers to their name, as they are not considered firms, in a strict and formal sense, for which reason they would not be allowed to subcontract the contracted works, as is shown in Figure 2.

Finally, the new regulation establishes a limitation on subcontracting firms (employment agencies) that solely supply workers to construction works for contracted activities (the “intensive subcontractor” concept). It all means that the subcontracting chain stops whenever reference is to a self-employed or to an “intensive subcontractor”. In other words, under no circumstances at all can a self-employed or an “intensive subcontractor” in turn subcontract.

In all cases, the duty of control over compliance with the inscription on the REA corresponds to the contracting firms that form part of the subcontracting chain, which are expected to require evidence from subcontractors of the REA Registration Certificate issued by the Administration.

## 2. Methodology

### 2.1. Data Collection

The prospective technique of the Survey [34,35] for optimization of the investigative process was used to request registration data on the REA from the different private construction sector firms. In addition, official databases were consulted, to obtain information relating to the registered entries on the REA referring to both types of Public Administrations: Municipal (Town and City) Councils and Provincial Councils (of Provincial Government). As a complementary means to the prospective study, the Qualitative Focus Groups Technique was used, with the objective of assuring the trustworthiness and the reliability of the data gathered in the surveys and to contrast the quality of the information [36].

### 2.2. Justification of the Choice of Public Administrations Under Study

The first phase of the research involved the study of the Autonomous Registry of Accredited Companies that is managed by each Autonomous Community and the Autonomous Cities or NUTS 2 level [37]. Although competency for its management corresponds to each Autonomous Community, its validity extends throughout Spanish national territory.

As the Public Administrations that are in closest contact with neighborhood communities provide most services and promote and manage most building works and infrastructure in Spain, a preliminary study was completed by analyzing governmental bodies and the organizational structure of second-level territorial divisions, which are the Provinces, the Balearic Islands and the Canary Islands and the Autonomous Cities of Ceuta and Melilla or NUTS 3 level [37].

The regional government deputation at provincial level or the *Diputaciones Provinciales*, as administrative structures with competency over the territory of the Province, and the Municipal (Town and City) Councils, with the closest area of reference to local communities, were selected for the purposes of the present study.

Both administrations have workers on their staff to provide services and to promote most of the construction works that meet the immediate needs of local communities. They therefore usually act as either contractors or subcontractors, which also means that their registration with the REA should be compulsory.

### 2.3. Study Design

In the present investigation, three universes of study were defined, as shown below, to assure greater understanding of the information of both a quantitative and a qualitative nature yielded by the different research techniques:

1. The data from the register were obtained through in situ and online consultation of the different databases of the 97 Public Administrations, the core of the present study. Data referring to 52 Municipal Councils and 45 Provincial Councils were obtained for the territorial area of the Kingdom of Spain (both corresponding to the NUTS 3 level).
2. A sample of SMEs from the construction sector, based in the Autonomous Communities of Castile-La Mancha and Castile-Leon, was obtained: 233 firms (106 firms from Castile-La Mancha and 127 from Castile-Leon). The size of the sample for both territories was computed by the following mathematical expression [38]:
  $$n=\frac{k^{2}*p*q*N}{\left(e^{2}*\left(N-1\right)\right)+k^{2}*p*q}$$
  where: *N*: Size of the population or universe (total number of possible interviewees); *k*: Constant that depends on the level of confidence that is assigned to the process under study. *e*: Desired sampling error. *p*: Proportion of individuals in the population with the characteristic under study. *q*: Proportion of individuals without that characteristic, in other words, 1-p. *n*: Size of the sample.
  The selection of Castile-La Mancha and Castile-Leon is because they are the two most similar Autonomous Communities by population, population density, and size of territory, as well as by the percentage of workers active in the construction sector and investment in training in matters of risk prevention in the workplace.
3. Focus Groups were designed to analyze the data obtained in the study and to provide feedback to the investigation, capable of arriving at conclusions and proposing possible measures for improvement. Two Focus Groups were prepared for that purpose (Table 3), one composed of construction sector experts and another of entrepreneurs from the construction sector, both with many years of experience.

## 3. Results

In the present study, the information referring to 97 Public Administrations (distributed between 52 Municipal Councils and 45 Provincial Councils) was analyzed, in relation to the data from 233 construction sector firms (106 from Castile-La Mancha and 127 from Castile-Leon). Likewise, the principal conclusions from the two Focus Groups of experts and entrepreneurs from the sector are shown. On the basis of their wide experience, the participants generated interesting conclusions, both justifying the results that were obtained and proposing improvements.

### 3.1. Municipal Councils

Following processing of the data that were collected, with regard to the Tax Identification Numbers of the 52 Municipal Councils selected for the study, their registration on the REA was verified and it was demonstrated that the very few Councils registered on the REA amounted to a mere 11.54% of the total (Table 4).

These data become worrying when the high level of non-compliance with this obligation to register on the REA is noted, which is the case of the Municipal Councils when they act as contractors in construction activities within the scope of their authority; an activity that is situated at values of almost 90.00%. Accordingly, this situation may be affirmed to be the outcome of highly demanding policies towards construction firms in the private sector and more relaxed ones towards public-sector administrations. Low compliance with this risk-prevention instrument is especially astonishing among Municipal Councils, administrations with significant construction activity in public works and services.

### 3.2. Provincial Councils

When examining the information collected with respect to the 45 Provincial Councils under analysis, it may be observed that the percentage results obtained were very similar to those shown in the study on Municipal Councils (Section 3.1).

A mere 15.56% of the Provincial Councils were compliant with their obligation to register. The level of non-compliance is concerning, because as many as 84.44% of Provincial Deputations in Spain failed to comply with the Law on Subcontracting (Table 5).

### 3.3. Private Construction Sector Firms

Upon analyzing the survey data, a clear change in tendency was observed contrary to the records obtained from the Public Administrations. The degree of compliance with registration on the REA by the private construction sector firms in the Autonomous Communities of Castile-La Mancha and Castile-Leon was higher than 90.00%.

If those values are analyzed in detail, the degree of compliance in the Autonomous Communities of Castile-Leon and Castile-La Mancha was 91.34% and 90.57%, respectively. Their levels of compliance with both registers can be categorized as very high (Table 6).

Moreover, the values of non-compliance among private sector construction firms can be qualified as very low, with non-registration rates close to 6–7%. Data were only registered from the 3% of firms that gave no response to the survey, for which reason their presence in the whole study was not significant and can be considered marginal.

The first evidence that arises from the data under analysis is the unequal degree of compliance that the Labor Authority tolerates with regard to the legal obligation of registration with the REA among firms from either the public or the private sector that are active in the construction sector.

### 3.4. Focus Group: Public Administrations vs. Private Firms

Two Focus Groups were designed, to test the results obtained in the prospective phase of the investigation: Group of Experts and Group of Construction Sector Entrepreneurs in Spain. The results of the organized debates were shown to the Focus Groups for the analysis of the registration with the REA of public and private, construction sector firms.

In absolute values, it was surprising that, of the 97 Public Administrations under analysis, only 13 (a mere 13.40%) were registered with the REA (precisely, six City and Town Councils and seven Provincial Councils), all the more so as it is an obligation established by the Law of Subcontracting, in order to be able to function as a contractor in the construction sector.

On the contrary, if these results are compared with those from the private sector firms, the degree of compliance is surprisingly very high, even higher than 90.00%. A result that is evidence of an alarmingly weak commitment within the Public Administration with regard to the enforcement of this administrative tool for preventive control, when public bodies should be an example of compliance with legal regulations, as they are applicable to all firms, whether public or private, without exception.

The conclusions from the debates of Focus Group 1: Experts (Table 7), confirmed a lack of commitment from the Public Administration in compliance with the applicable legal requirements, in contrast with the rigor that the Labor Authority shows towards firms that have no public affiliation. It is evidence of bad practice and is a bad example for private sector construction firms, as well as evidence of non-compliance with current legality by Public Administrations engaged in construction activities.

The experts from Focus Group 1 argued that the firms from the private sector that are not registered with the REA are active in the so-called “underground economy”, with workers not on the Social Security register and with work centers in dire need of safety measures. They likewise recognized the difficulty when prosecuting non-declared works completed by firms with no REA registration, as no action is normally taken unless a complaint is submitted. As a corrective measured, increasing the number of inspections from the Labor Authority was proposed, through campaigns directed at prosecuting the ‘underground economy’, and requiring the commitment of the Public Administrations to review registration status on the REA and to reverse that situation in the future.

The principal conclusions of the discussion in Focus Group 2: Entrepreneurs (Table 8) are centered on the excessively lengthy waiting periods for registration with the REA, which differ by Autonomous Community registry, lasting days or even months from the time of registration until formalization of all information or attachment of complementary documentation as required.

There was likewise coincidence over the blame placed on the Labor Authority for not intensifying inspections, with the objective of prosecuting non-compliant firms and to avoid unfair competition with regard to other firms that are indeed registered.

In the debate, the high compliance of private sector firms stood out, with values that surpassed 90.00%. However, it was said that many firms formally met the requirement for registration with the REA, more out of a fear of economic sanctions than because of the conviction that registration has to be done, to guarantee safe working conditions both for the workers and for the work centers.

In conclusion, the participants of both Focus Groups voiced their surprise at the results, as revealing as much as they are alarming, in so far as they demonstrate the shallow commitment of the Public Administrations, due to their non-compliance with what is in fact a requirement for private sector firms. Equally, both Focus Groups coincided in so far as the future is hopeful and that working alongside the Labor Authority, the public-sector situation can be reversed and the private sector data improved.

## 4. Conclusions

Based on the analysis of the results, it can be affirmed that the Public Administrations in Spain are in large measure non-compliant with the obligation to register on the Registry of Accredited Companies (REA) (Table 2), when acting as contractors in the construction sector. The registration rates of only 13.40% among Public Administrations (Table 4 and Table 5) can be contrasted with the data from the private sector firms where registration stood at over 90.00% (Table 6).

The seriousness of this act of non-compliance is evident, because it is an administrative requirement for risk-prevention and control established under Law 32/2006, regulating subcontracting activities in the construction sector in Spain for all firms, including both public bodies, Municipal Councils and Provincial Councils, when acting as contractors in construction, in the field of their competence.

These circumstances are evidence of a certain slackness in the “*modus operandi*” of the Public Administrations, which are accompanied by information practices that are not transparent and a lack of commitment towards compliance with the Law. This division generates a feeling of impunity towards the rest of society, as well as a clear comparative abuse with regard to the firms that form part of the private sector. This “bad example” might mean that firms from the private sector believe that it is merely a further administrative requirement (a mere documentary formality), overlooking the fact that it is a risk-prevention tool that seeks to guarantee worker safety.

Both Focus Groups (Table 7 and Table 8), organized in order to debate this behavior of the Public Administrations that are engaged in construction activities, were very enlightening. In many cases, remarkably similar opinions to the opinions of the Experts and Entrepreneurs from the construction sector were expressed, among others, the following:

- Incompliance by the Public Administrations engaged in construction activities with the duty to register with the REA, as required by Law, is a bad example for society. The behavior of the Public Administrations should be exemplary and a model to follow for other institutions and the public. This type of non-compliance results in less protection for the workers, which imply weaker guarantees for their safety and health.
- Trades union organizations do little to challenge these types of behavior, for which reason they should be more combative, demanding compliance with the Law from the Public Administrations. This lack of commitment is partly due to unawareness within the Trades Unions of non-compliance with the REA among the Public Administrations. Through this study, therefore, the aim is to bring that reality to the forefront.
- The Labor Inspectorate should follow up Public Administrations that are not compliant, obliging compliance with the law by sanctioning inappropriate behaviors. One of the measures that the construction sector demands is to be able to cross-check data and information between the different Public Administrations that are involved, as well as prosecuting and increasing fines imposed on illegal construction sites (that have not communicated the opening of a workplace).
- Increasing the number of inspections is a coercive measure to prosecute legal incompliance among both public and private firms, sanctioning those behaviors and demanding compliance with the Law.

It is more than evident that the non-compliance of the Public Administrations, as well as contravening the contents laid down in Law 32/2006, in Regulation of Subcontracting Activities in the construction sector in Spain, implies a somewhat unexemplary conduct for society and its citizens, imposing requirements on the firms from the private sector which in turn are not demanded of the firms with construction activities in the public sector.

As a final conclusion, and despite the preventive actions conducted by the Administration to improve and to guarantee worker safety and to integrate prevention in the construction processes, there are still aspects that must be improved. One clear example is the control exercised over firms that act as contractors and subcontractors in construction activities, because the Labor Inspectorate should enforce specific requirements on all firms seeking to obtain REA certification, including those in the public sector.

It will indeed be necessary to move in that direction, as launching risk-prevention instruments without mechanisms for supervision and follow up means having blunt and ineffectual instruments for control over incidence rates that blight the construction sector and, despite all administrative efforts, continue to increase year in year out.

## Figures and Tables

**Table 1:** Spanish construction firms, classified by size, in accordance with the number of workers regulated in Recommendation 361/2003 of the European Commission over the period between 2008 and 2017. *Source:* Dirección General de Industria y de la Pequeña y Mediana Empresa [20].

Size of Construction Firm	2008	%	∑%	2015	%	∑%	2017	%	∑%
**Micro-firms (1–9 emp.)**	574,663	92.38	**99.18**	393,192	96.88	**99.70**	395,902	96.10	**99.69**
**Small (10–49 emp.)**	42,275	6.80		11,463	2.82		14,801	3.59	
**Medium (50–249 emp.)**	4424	0.70	**0.82**	1014	0.25	**0.30**	1225	0.29	**0.31**
**Large (250 emp. or more)**	734	0.12		180	0.05		62	0.02	
**Total**	622,096	100.00	100.00	405,849	100.00	100.00	411,990	100.00	100.00

**Table 2:** Extract from the “Frequently Asked Questions” (FAQ) from the REA software application. *Source:* Ministerio de Trabajo, Migraciones y Seguridad Social [30].

Question	Answer
Does each Autonomous Region have a Registry of Accredited Companies?	Yes. Each Autonomous Region, as well as Ceuta and Melilla, has to establish its own Registry of Accredited Companies, which will depend on its respective Labor Authority.
Where is the Registry inscription valid?	Inscriptions on the REA will be valid across all national territory.
Who is obliged to register?	All firms and self-employed workers with salaried workers that assume the role of contractor or subcontractor for the completion of works on a construction site have to be registered with the REA. Registration with the REA in no way exempts the registered firm from the obligation, whenever so required by the Labor Authority, of justifying its compliance with the requirements contemplated under article 4, Sections 1 and 2a) of Law 32/2006, of October 18th, in Regulation of Subcontracting in the Construction Sector.
Do Public Administrations, for example, Municipal Councils also have to register?	Yes, the Administrations have to appear on the registry, if they participate as contractors or subcontractors in the process of subcontracting in the construction sector.

**Table 3:** Position of the intervening parties/experience in years, duties of the moderator, debate content and duration. *Source:* Mind the Gap: Professionalization is the Key to Strengthening Safety and Leadership in the Construction Sector [39].

Parties		Moderator	Content/Time		
Provincial Director of the Labor Inspectorate and Social Security (10 years). Provincial Director of Health and Safety at Work Service (14 years). Risk-Prevention Specialist at the Spanish Confederation of Business Organizations (9 years). Area Manager at the Construction Labor Foundation (22 years). Risk-Prevention Specialist at the Construction Labor Foundation (8 years). Director of an ORP company (12 years). Legal advisor of a professional college of technical architects and building engineers (21 years). Head of the Risk-Prevention Service of a large Spanish company with over 250 employees (12 years).		Propose the topics to be addressed. Promote participation and desire for discussion. Moderate the progress of the interventions and discussion, so that it remains relevant. Provide statistical data from national and European studies concerning the topics to be addressed.	Regulatory framework which operates in the construction sector and characteristics of the sector. Registration of the Public Administrations and private sector firms with the REA. Complexity of the sector, associated risks and their assessment, preventive structures. Consultation and participation.		
**Focus Group 1: Experts**					
Total:	**8**				**150 min**
Company specializing in renovations and restorations (30 years). Company specializing in waterproofing (12 years). Company specializing in new constructions and renovations (15 years). Company specializing in civil and building works (18 years). Company specializing in public works (20 years). Company specializing in the assembly and rental of scaffolding (25 years). Company specializing in electricity (22 years).			Registration with the REA of the Public Administrations and private sector firms. Difficulties in integrating risk prevention in their companies, in accordance with the reference standards in force. Personal opinion of the content set out in Focus Group 1.		
**Focus Group 2: Entrepreneurs**					
**Total:**	**7**	**1**			**120 min**

**Table 4:** Municipal Councils in the present research registered with the REA, values updated until 02/13/2017. Where: 100% = 52 Municipal Councils in national territory. *Source*: Ministerio de Hacienda y Función Pública. Inventario de Entes del Sector Público Local [40].

Public Administration	REA Registered Firms	%	Firms not Registered with the REA	%	Total	∑%
**Municipal Councils**	6	**11.54**	46	**88.46**	52	100.00

**Table 5:** Registrations on the REA by the Provincial Councils under study, values updated until 02/13/2017. Where: 100% = 45 Provincial Deputations in national territory. *Source*: Ministerio de Hacienda y Función Pública. Inventario de Entes del Sector Público [40].

Public Administration	REA Registered Firms	%	Firms not Registered with the REA	%	Total	∑%
**Provincial Councils**	7	**15.56**	38	**84.44**	45	100.00

**Table 6:** Registration with the REA by Private Firms from the sector under study until 13/02/2017. Where: 100% = 106 Firms from Castile-La Mancha, and 100% = 127 Firms from Castile-Leon; the sample under analysis amounts to 233 Firms from the construction sector active in the national territory. *Source*: Ministerio de Trabajo, Migraciones y Seguridad Social [30].

Autonomous Communities	REA Registered Firms	%	Firms not Registered with the REA	%	Don’t Know/No Opinion	%	Total	∑%
**Castile-La Mancha**	96	**90.57**	7	6.60	3	**2.83**	106	100.00
**Castile-Leon**	116	**91.34**	7	5.51	4	**3.15**	127	100.00

**Table 7:** *Focus Group 1: Experts.* Conclusions on registration with the REA by Public Administrations and by private sector firms, complemented by measures for improvement.

Reference Indicators	Debate	Improvement Proposals
Only 13.40% of the Public Administrations are registered on the REA.	High non-compliance with the duty of registration with the REA among Public Administration firms.	The Administration that is not upholding the Law is a bad example for society.
		Greater responsibility of the Public Administrations towards construction activity is necessary.
No sanctions of Public Firms registered by the Labor Authority.	Unequal treatment of Private Construction Firms in comparison with Public Construction Firms.	The Labor Authority should conduct more inspections of construction firms linked to the Public Administrations.
		The Trades Union representatives of the Public Administration should involve themselves by demanding compliance with the Law.
Over 90.00% of construction firms from the private sector comply with REA registration.	High indices of responsible behavior of construction firms in the Private sector.	It is important to carry out more inspections to prosecute non-compliant firms (underground economy).
	The REA has increased the solvency and quality of construction sector firms.	It would be more efficient to unify the administrative processes for registration with the REA throughout all of the Autonomous Communities of Spain. The Labor Authority should have sufficient material and human resources to intensify inspection work.
	The REA is a good instrument to control subcontracting in the construction sector.	

**Table 8:** *Focus Group 2: Entrepreneurs.* Conclusions on registration with the REA among Public Administrations and among private sector firms, complemented by improvement measures.

Reference Indicators	Debate	Improvement Measures
Only 13.40% of Public Administrations are registered with the REA.	A lack of commitment is evident from the Public Administrations.	Public Administrations should comply with the Law and register their firms with construction activity with the REA.
		The Public Administrations should be an example for the public.
No sanctions against Public Firms have been registered by the Labor Authority.		Greater social commitment is necessary from politicians and Public Institutions.
		The requirement for REA registration should be included in the Law on Public-Sector Contracts.
Over 90.00% of private sector construction firms comply with REA registration.	We should reach 100% compliance of private sector construction firms.	Work inspections should be intensified to prosecute non-compliant firms with unfair competitive practices (underground economy).
	Administrative procedures for registration with the REA should be simplified.	They should unify the documental requirements for registration with the REA in all the Autonomous Communities of Spain.
	It is possible that a large number of the construction firms register with the REA rather than face fines and sanctions.	Corporate commitment from the directors and managers of the construction firms towards risk prevention and safety at work is necessary.
	Registered firms with safety regulations in place should receive incentives.	The commitment of firms towards the safety of their workers should be incentivized with a reduction in social security overheads.
